# Key recruitment and retention strategies for a pilot web-based intervention to decrease obesity risk among minority youth

**DOI:** 10.1186/s40814-019-0492-8

**Published:** 2019-09-05

**Authors:** Grisselle DeFrank, Sarina Singh, Katrina F. Mateo, Laura Harrison, Alyson Rosenthal, Allison Gorman, May May Leung

**Affiliations:** 1 Hunter College School of Urban Public Health, City University of New York (CUNY), 2180 Third Avenue, New York, NY 10035 USA; 2 CUNY Graduate School of Public Health and Health Policy, 55 W 125th Street, New York, NY 10027 USA; 3 Children’s Aid, 711 Third Avenue, Suite 700, New York, NY 10017 USA; 4 Weill Cornell Medical College, 407 East 61st Street, New York, NY 10065 USA

**Keywords:** Childhood obesity, Research methods, Preadolescents, Trial, Community, Race/ethnicity, Recruitment, Retention, Pilot

## Abstract

**Background:**

Interactive Nutrition Comics for Urban Minority Youth (*Intervention INC*) is an innovative, web-based interactive comic tool for dietary self-management, which aims to decrease obesity risk among urban minority preadolescents. The feasibility and acceptability of Intervention INC was assessed by implementing a two-group randomized pilot study. To date, intervention studies have typically faced various barriers in recruiting and retaining study participants. The purpose of this paper is to describe recruitment and retention activities from this study and in particular, discuss challenges faced, strategies implemented, and lessons learned.

**Methods:**

Black/AA and Latino children (ages 9–12 years) and their parent/guardian were recruited from East Harlem/Harlem, New York. Recruitment strategies included flyering in the community, having a convenient study location, providing participation incentives, and partnering with community/school-based organizations. Potential participants were screened for eligibility; enrollees completed online surveys and interviews at baseline (T1), intervention midpoint (T2), intervention end (T3), and 3-months post-intervention (T4). Retention strategies included flexible scheduling, reminder calls/texts, incremental compensation, and consistent study staff.

**Results:**

Eighty-nine enrolled dyads completed a T1 visit (August to November 2017) and were randomized to the experimental (*E*, *n* = 45) or comparison (*C*, *n* = 44) group. Enrolled dyads learned about the study through community events (39%), community flyering (34%), friend/referral (15%), or a community clinic partner (12%). T1 child demographics were mean age = 10.4 ± 1.0 years, 61% female, 62% Black and 42% Latino, and 51% overweight/obese; parent demographics were mean age = 30.8 ± 8.9 years, 94% female, and 55% Black and 45% Latino. Survey completion rates by dyad were high throughout the study: T2, 87%; T3, 89%; and T4, 84%. Average data collection per session was 65 min. Parents at T4 (*n* = 76) felt they received enough study information (97%) and that their questions were answered properly (80%). Eighty-one percent of children at T4 (*n* = 75) were very satisfied/extremely satisfied with how study staff communicated and interacted with them.

**Conclusion:**

Effective recruitment strategies consisted of community events and flyering, while a variety of retention strategies were also used to successfully engage urban Black/AA and Latino families in this study. Though our findings are limited to only Latino and Black families in low-income neighborhoods, we have identified successful strategies for this specific high-risk population and potentially similar others.

**Trial registration:**

ClinicalTrials.gov, NCT03165474, registered 15 May 2017

## Background

Childhood obesity is an ongoing public health crisis in the United States (US) that disproportionately affects low-income, minority individuals. A 2013 study showed that black and Hispanic children, compared with non-Hispanic white children, have substantially higher body mass index (BMI) *z*-scores, total fat mass index, and prevalence of overweight and obesity [1]. Interventions delivered via web-based or mobile platforms (mHealth) that incorporate tailored and/or engaging health promotion content and utilize theory-driven strategies may help at-risk children and their parents improve their dietary-related practices to lower risk for childhood obesity and improve health outcomes. Importantly, however, rigorous evaluation of these interventions is needed to demonstrate their feasibility for delivery and adoption in real-world settings.

To date, intervention studies have typically faced numerous barriers in recruiting and retaining study participants, especially within at-risk populations. A well-documented challenge of minority recruitment is distrust of research, and especially clinical research [2–4]. Distrust stems from a history of discrimination as well as notorious research abuses, such as the US Public Health Service Tuskegee Syphilis Experiment [5]. Other participant barriers include lack of transportation, scheduling conflicts, recruitment materials at inappropriate literacy levels, and lengthy consent forms [6]. Further barriers on the part of researchers include a lack of understanding about cultural differences among ethnic minorities and subsequent poor communication with these groups at all research stages [6].

Recruiting and retaining minority children specifically for obesity-related interventions pose several additional challenges. A 2012 study by Wright et al. focused on the impact of a school health program on physical activity and BMI in minority children. The authors acknowledged that a major limitation of the study was retention, especially at 12-month follow-up [7]. Wright et al. suggested that a potential retention barrier was a lack of incentives, which could have inhibited parents who had to rush home after work to attend the program. In addition, child participation in studies requires significant family involvement, as parents, guardians, or caregivers must consent, provide support, and coordinate children’s participation, actions, and responses. Any barriers to parents/guardians and families, therefore, can reduce successful child retention [8]. Another possible challenge to retaining minority children is maintaining their engagement throughout entire interventions [8]. It is therefore important for study staff to use creative tactics and planning strategies to ensure that children do not lose interest in the studies.

To date, few studies have attempted to analyze information about recruitment and retention in childhood obesity-related health interventions upon their completion [9, 10]. A 2015 systematic review by Cui et al., which analyzed recruitment and retention strategies of studies involving low-income, minority children, noted that one third of eligible studies had not a published peer-reviewed paper [8]. This highlights missed opportunities to report on the success or failure of recruitment and retention strategies used for minority children. In addition, well-documented rates of high attrition have led to limited interpretability and generalizability of study findings [11–13]. It is therefore important to identify key strategies that can lead to successful recruitment and high retention rates of minority children in intervention studies but also report on the implementation and impact on retention.

Therefore, the purpose of this paper is to describe recruitment and retention activities from the Interactive Nutrition Comics for Urban Minority Youth (Intervention INC) study (a 4.5-month pilot RCT), strategies implemented in response to challenges during study implementation, as well as discuss lessons learned and implications for similar intervention studies, particularly with minority youth.

## Methods

### Pilot study design

Intervention INC was a two-group randomized pilot study that assessed the usability, feasibility, and acceptability of an innovative, web-based interactive comic tool aimed at decreasing obesity risk among urban minority preadolescents. The study protocol and intervention are described briefly below, and in greater depth elsewhere [14].

Participants were recruited from low-income NYC neighborhoods from August to November 2017. Child-parent dyads were eligible if the child was 9–12 years old at baseline, identified as either Black/AA or Latino, had a BMI percentile of 5% or higher, and was able to speak and read in English, and the parent was able to read and speak in either English or Spanish and was primarily responsible for providing or purchasing food for the child. Dyads also had to have regular internet access via a tablet device, smartphone, or computer/laptop with internet access. Both children and parents were compensated for time to complete questionnaires/interviews and travel to in-person study visits with gift cards (in increasing amounts for each data collection time point) and round-trip Metrocards. Parent consent and child assent were obtained prior to initiation of any study procedures.

For the intervention, children in the experimental group had access to a 6-chapter interactive nutrition comic (one chapter released a week), a goal-setting and self-assessment feature, and weekly text/email messages and reminders from comic characters, while children in the comparison group had access to six online newsletters with nutrition-related content, a goal-setting and self-assessment feature, and weekly text/email messages and reminders. Parents in both the experimental and comparison groups received six online newsletters with healthy feeding-related content and weekly text/email messages in reminders, but experimental group parents also had access to the child nutrition comic.

Dyads participated in the study for 4.5 months, which included a 6-week intervention and 3-month follow-up. Data collection occurred at four time points: baseline (T1), intervention midpoint or 3-weeks post-baseline (T2), intervention end or 6-weeks post-baseline (T3), and 3-month follow-up post-intervention (T4). T1 and T4 time points were in-person study visits, while T2 and T3 were conducted via phone and online. At T1, the child’s height, weight, and body composition were measured. Both the child and parent then completed individual baseline questionnaires on laptop devices, which collected demographics and dietary-related outcome data. For the child, these data related to dietary knowledge, attitudes, and intake. For the parent, these data related to feeding practices and the home food environment [14]. Similar questionnaires were administered at T2 (child-only), T3 (child and parent), and T4 (child and parent). In addition to questionnaires, children and parents also completed semi-structured interviews to collect data about their experiences using the website at T2 (child-only), T3, and T4. At T4, children had their height, weight, and body composition taken again. Both the children and parents also completed additional questionnaire items related to the overall study participation experience.

### Recruitment protocol

#### Initial recruitment protocol

Through a partnership with a child-focused community-based organization (CBO) focused on serving at-risk youth in the East Harlem/Harlem neighborhoods of NYC, study recruitment letters were sent to parents/guardians of children who had utilized services from their community health clinic within the previous 2 years. Using basic child inclusion/exclusion criteria (age 9–12 years and BMI percentile above 85%), a list of potential participants was generated, from the CBO’s existing patient population, with the child’s contact information. Bilingual recruitment letters signed by the CBO Medical Director, a CBO Pediatrician, and the study Principal Investigator were sent to the parents/guardians of these children. The letters contained a brief description of the study, incentives, and contact information of study staff. Interested individuals had the option of calling, texting, or emailing study staff for additional information. These parents were also called by study staff approximately 1 week after letters were sent to assess interest in study participation. Study staff screened interested individuals and scheduled T1 visits. As participants were enrolled and scheduled for T1 visits, study staff sent reminder texts, calls, and emails (as preferred by participants) three days and one day prior to appointments.

#### Changes to recruitment protocol

Initial challenges with recruitment and enrollment led to the broadening of our recruitment approaches. Specifically, the BMI percentile criteria were changed from 85 to 5% [14]. This BMI range includes healthy, overweight, and obese children. Following this change in inclusion criteria, updated potential participant lists from our CBO partner (i.e., with widened BMI percentile criteria to above 5%) were created and additional recruitment letters were sent to families not included in the first round. Furthermore, our recruitment protocol was expanded to include flyering 3–5 days a week in East Harlem/Harlem neighborhoods. Study staff compiled a list of key recruitment outreach locations and conducted targeted flyering in these areas, which included churches, medical offices, housing complexes, schools, community organizations, and recreational spaces. To keep track of the areas covered, an interactive Google map was created and updated weekly by study staff. In addition to neighborhood flyering, we partnered with community organizations and local elementary/middle school schools that permitted study staff to table at their weekend/after-school events. At these events, study staff engaged youth in a fun, nutrition card game while providing parents with information about the study. Potential participants were screened in-person at the events to expedite enrollment.

#### Data collection

Once parents expressed interest in joining the study, study staff screened subjects for eligibility by assessing inclusion and exclusion criteria such as the following: Does your child read and speak English? Do you have access to an iPad, tablet, desktop, laptop, or phone with texting capabilities with internet access? Potential dyads received numerical IDs. Study staff monitored appointments via a Google Calendar accessible to all study staff using these IDs. At least 1 member of the study staff was responsible for monitoring each potential dyad and followed up with any potential dyad contact within 24 hours. If the parent was Spanish-speaking, a bilingual study staff member would be assigned to that dyad.

Study staff kept track of weekly recruitment/enrollment status, all communications with dyads, and number of dyads enrolled per week. Specifically for every dyad, the following information was collected during recruitment: who made first contact (participant or study staff), mode of contact, date of first contact, study staff that communicated with participant, number of contact points until scheduled, how they learned about the study, race, ethnicity, and age of child. In addition, study staff held weekly meetings to discuss these outcomes and develop improvement strategies as challenges surfaced. We anticipated to recruit 82 study dyads over the course of 3 months.

During T1, dyads completed demographic surveys in person at the Hunter College campus located in East Harlem. Surveys were administered as a questionnaire through a laptop computer in a private room with parent, child, and 2 research study staff. Participants were told that they should complete the surveys individually and study staff were available to assist. Challenges were systematically documented and discussed during weekly research team meetings, and strategies were implemented to address any challenges.

### Retention protocol

Study staff offered flexible scheduling 7 days a week, which included after-school and work hours. At the end of each T1 visit, study staff documented expected follow-up dates for the midpoint, endpoint, and 3-month follow-up visits for the participants. As each time point approached, the date and time of the following visit or follow-up were confirmed and/or adjusted, and updated on the Google calendar. Once appointments were confirmed, study staff conducted 3-day and same-day reminder calls or texts; and a 1-day confirmation text or call. Visit outcomes were labeled as completed, rescheduled, or no-show. If dyads were 10 min late to their appointment, they were contacted by study staff. After 30 min, dyads were considered “no-shows” and study staff immediately reached out to request a reschedule.

To maintain dyad comfort and consistency with the research project, one study staff was assigned to each dyad so that the dyad interacted with the same study staff member through the study period and at each time point. Dyads were also sent newsletters and recipes between T3 and T4 to encourage continued engagement in the study. Thank you cards were sent at T3 with compensation for completing T3, a reminder of the upcoming appointment in 3 months, and appreciation of continued participation.

#### Incentives

Participants were compensated with round-trip MetroCards for in-person data collection and gift cards of their choice for each time point. Gift card options included the following: large department store, supermarket chain specializing in selling organic products, discount supermarket chain, and sporting goods store. Selection of these gift cards was informed by formative research asking parents and input from a community-based organization (not published). Gift card monetary value increased at each time point: at T1, children received $10 gift cards and parents received $15 gift cards; at T2, children received $15 gift cards; at T3, both children and parents received $15 gift cards; at T4, children received $25 gift cards and parents received $30 gift cards. Participants were also informed that upon completion of all time points, they would be entered into a raffle to win a $100 gift card. To enhance the study experience, participants were provided healthy snacks and beverages (tea, coffee, water) at any in-person study visits.

#### Data collection

Study staff logged completion dates of each time point, durations of various measures (e.g., time taken to complete surveys), notes on dyad communication, dyad status, and retention percentages on a shared spreadsheet. In addition, barriers to website access (e.g., technological, password, and scheduling) and any comments about the study that participants communicated to study staff (not as part of data collection) were noted in the shared spreadsheet. Dyads were considered lost-to-follow-up if they did not respond to all attempted methods of communication from study staff including text, email, phone call, and mail within 2 weeks after their scheduled T2 or T3 visit or within 4 weeks after their scheduled T4 visit. A dyad was considered to have completed a time point if: both parent and child completed surveys at T1, T3, and T4. This information was organized into a shared spreadsheet that was regularly updated and reviewed at study staff meetings. Strategies for retention were reviewed and amended as needed.

At T4, children and parents individually completed study experience surveys. All questions were asked on a Likert scale from 1 (not satisfied) to 4 (extremely satisfied). Questions for guardians included the following: How satisfied were you with your experience in this study? Did we give you enough information about the study? How easy was it to get in touch with us if you had a question? Questions for children included the following: How satisfied were you with your experience in this study? How satisfied were you with how we communicated and interacted with you?

### Data analysis

Data was analyzed using IBM SPSS Statistics for Windows, Version 22.0. Armonk, NY: IBM Corp. Descriptive statistics were run for all demographic variables for enrolled participants. Chi-square tests were used to compare demographics of experimental and comparison arm participants, with a significance level of *p* = 0.05. Retention data were analyzed using binary logistic regression to assess characteristics of participants who completed each data collection point compared to those who did not.

Study protocols and detailed study notes documented by study staff were systematically reviewed to identify significant changes, adjustments, or barriers to implementing the original protocol. Implementation of key recruitment and retention strategies that were documented throughout study implementation were also systematically reviewed with team members and reflected upon.

## Results

### Recruitment

Recruitment occurred between August 2017 and November 2017. Key recruitment strategies implemented included flyering, partnering with a community clinic, and attending community events. Table 1 shows recruitment activities by month.

**Table 1:** Intervention INC recruitment activities by month

Month	Activities
August	• Mailed recruitment letters to individuals that met initial inclusion criteria • Posted flyers in 25-block radii in East Harlem • Participated in first community event (a “back to school”-themed celebration) where children and parents played nutrition games and received information about the study
September	• Sent reminders for T2 appointments via texts and phone calls • Handed out flyers at large shopping complexes, local businesses, and other areas with high foot traffic • Participated in fall- and school-themed events where information about the study was provided
October	• Sent reminders for T3 appointments via texts and phone calls • Re-posted flyers in 25-block radii in East Harlem • Participated in community events at new locations such as food-access organizations and local elementary schools; they also began to screen potential participants at community events to expedite enrollment
November	• Mailed second batch of recruitment letters to potential participants • Provided flyers to churches

#### Enrollment by activity

**Fig. 1:**
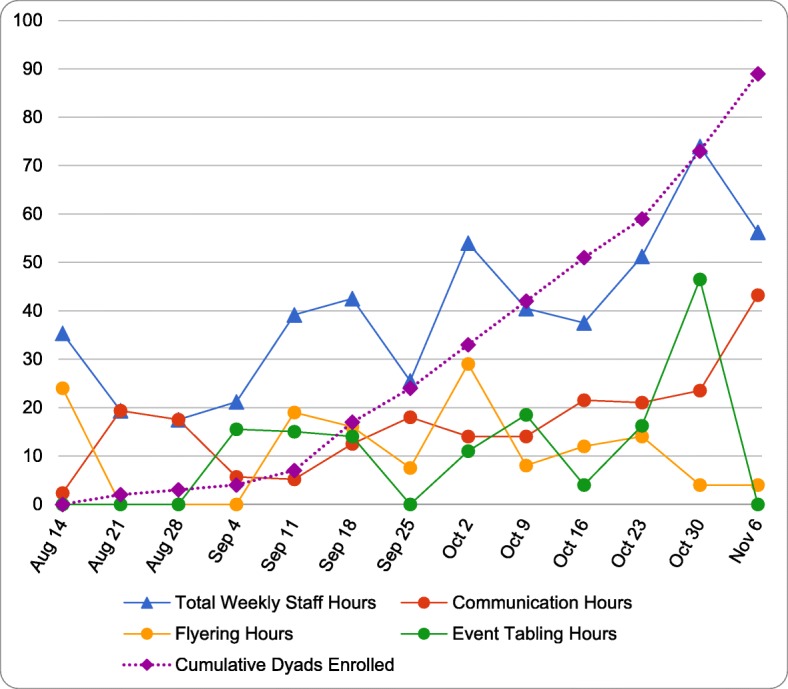
Intervention INC recruitment activity hours and dyads enrolled

Figure 1 shows the total number of hours spent on recruitment per week (triangular points), the number of hours spent on each recruitment activity per week (circular points), the number of dyads enrolled per week (diamond points) as a cumulative number (triangular points). Study staff spent 514 total hours recruiting 89 dyads. Overall, weekly staff hours increased from the week of August 14th (35.33 h) to the week of November 6th (56.25 h). Number of dyads enrolled weekly also increased from August 14th (0 dyads) to November 6th (16 dyads). In addition, the rate of dyads enrolled increased after September 11th and again after October 23rd. Weekly communication hours (texting, calling, emailing, and mailed letters) declined from the week of August 28th (17.5 h) to the week of September 11th (5.17 h) but had an overall increase from the week of September 11th (5.17 h) to the week of November 6th (43.25 h). From the week of October 23rd to the week of October 30th, there was a large increase in event tabling hours as well as number of dyads enrolled. Weekly flyering hours fluctuated considerably for the duration of recruitment.

#### Participant enrollment details

**Fig. 2:**
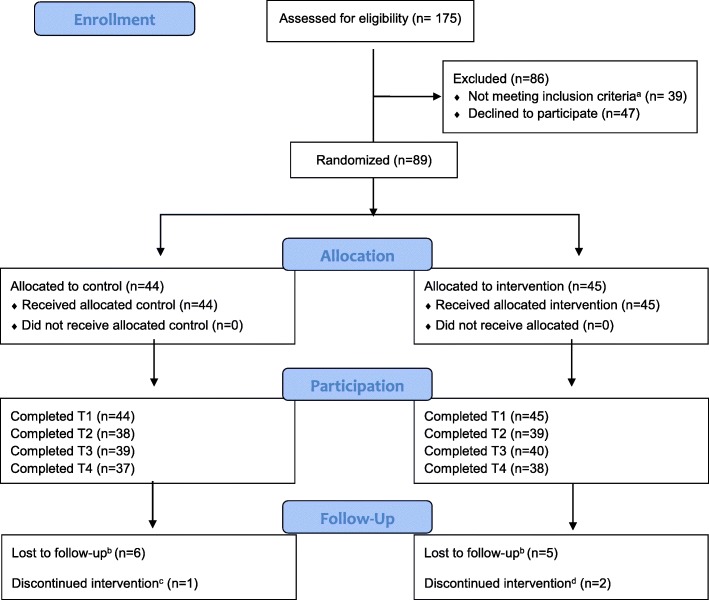
CONSORT diagram: intervention INC pilot study. a Did not meet study criteria due to heart condition, BMI, parent did not speak Spanish or English, did not have internet access, race/ethnicity reading problem, attendance and/or, age. b No response to communication attempts including calls, emails, texts or mailings. c Parent was unable to continue study due to medical reasons. d Child did not want to continue participating in the study

The CONSORT diagram in Fig. 2 shows the progress of study recruitment. One hundred and seventy-five people (adults) were initially screened for eligibility, and of these, 47 declined to participate and 39 did not meet the inclusion criteria due to (categories not mutually exclusive) age (20); race/ethnicity (7); medical condition, e.g., heart condition; reading or learning disability (21); attendance issues (12); and access to technology (5). Of the 89 participants that were enrolled in the study, 30 learned about the study through flyering, 11 through the partner community clinic (includes cold calling and letters), 35 through community events that were staffed by trained study staff, and 13 through a friend/referral. One hundred and thirty-three parent-child dyads were scheduled for a baseline appointment, 89 dyads completed a baseline visit, and were randomized. The last participant was enrolled on November 11, 2017, for a total recruitment period of 4 months.

#### Baseline characteristics of enrolled participants

A total of 89 parent-child dyads were recruited into the study. Table 2 shows the characteristics of the adult and child participants enrolled into the study. The demographic breakdown of the adults enrolled are mean age 30.8 ± 8.9 years; 94.4% female; 3.4% White/Caucasian, 50.6% Black/African American, and 39.3% Hispanic/Latino; 73% U.S. born; 20.5% less than high school; and 60.2% of adult participants participated in SNAP. The demographic breakdown of the children enrolled are mean age 10.4 ± 1.0 years, 59.3% female, 46.2% Black/African American and 29.7% Hispanic/Latino, and 97.8% U.S. born.

**Table 2:** Demographic characteristics of Intervention INC participants

	Overall *N* (%)	Experimental *N* (%)	Comparison *N* (%)
Parent			
Age			
< 35	28 (31.5)	15 (33.3)	13 (29.5)
36-45	37 (41.6)	19 (42.2)	18 (40.9)
46–55	21 (23.6)	10 (22.2)	11 (25.0)
55+	3 (3.4)	1 (2.2)	2 (4.5)
Gender			
Male	5 (5.6)	2 (4.4)	3 (6.8)
Female	84 (94.4)	43 (95.6)	41 (93.2)
Race/ethnicity			
White/Caucasian	3 (3.4)	1 (2.2)	2 (4.5)
Black/African American	45 (50.6)	21 (46.7)	24 (54.5)
Hispanic/Latino	35 (39.3)	18 (40.0)	17 (38.6)
Multiracial/other	6 (6.7)	5 (11.4)	1 (2.3)
Household income**			
< $20,000	29 (32.6)	15 (33.3)	14 (32.6)
$20,000–$39,999	30 (33.7)	17 (37.8)	13 (30.2)
$40,000–$59,999	18 (20.2)	9 (20.0)	9 (20.9)
$60,000 or more	11 (12.4)	4 (8.9)	7 (16.3)
Country of birth			
USA	65 (73.0)	32 (71.1)	33 (75.0)
Foreign born	24 (27.0)	13 (28.9)	12 (27.3)
Highest level of education**			
Less than HS/finished HS/GED	28 (31.8)	17 (37.8)	11 (25.6)
Some college/finished college	52 (59.1)	24 (53.3)	28 (65.1)
Other	8 (9.1)	4 (8.9)	4 (9.3)
Marital status**			
Single	40 (45.5)	23 (51.1)	17 (39.5)
Married/in marriage-like relationship	34 (38.6)	16 (35.6)	18 (41.9)
Separated/divorced/widowed	14 (15.9)	6 (13.3)	8 (18.6)
Relationship to child**			
Mother/stepmother	78 (88.6)	42 (93.3)	36 (83.7)
Father/stepfather	4 (4.5)	1 (2.2)	3 (7.0)
Grandmother	4 (4.5)	1 (2.2)	3 (7.0)
Other	2 (2.3)	1 (2.2)	1 (2.3)
SNAP participation**			
Yes	53 (60.2)	27 (60.0)	26 (60.5)
No	35 (39.8)	18 (40.0)	17 (39.5)
I don’t know			
Child			
Age			
9–9.99 years	23 (25.8)	11 (24.4)	12 (27.3)
10–10.99 years	22 (24.7)	12 (26.7)	10 (22.7)
11–11.99 years	31 (34.8)	16 (35.6)	15 (34.1)
12–12.999 years	13 (14.6)	6 (13.3)	7 (15.9)
BMI class			
Normal	42 (47.2)	21 (46.7)	21 (47.7)
Overweight	19 (21.3)	9 (20.0)	10 (22.7)
Obese	28 (31.5)	15 (33.3)	13 (29.5)
Gender			
Male	35 (39.3)	17 (37.8)	18 (40.9)
Female	54 (60.7)	28 (62.2)	26 (59.1)
Race/ethnicity***			
Black only	42 (47.2)	20 (44.4)	22 (50.0)
Hispanic only	29 (32.6)	15 (33.3)	14 (31.8)
Black and Hispanic	8 (9.0)	6 (13.3)	2 (4.5)
Mixed—Black	6 (6.7)	3 (6.7)	3 (6.8)
Mixed—Hispanic	4 (4.5)	1 (2.2)	3 (6.8)
Country of birth			
USA	87 (97.8)	43 (95.6)	44 (100.0)
Foreign born	1 (1.1)	1 (2.2)	0 (0.0)
Did not know	1 (1.1)	1 (2.2)	0 (0.0)

*No significant differences were found between Experimental and Comparison for demographic characteristics

**One participant did not answer the survey question

***Race/ethnicity not mutually exclusive

#### Recruitment strategies and challenges

Table 3 summarizes the recruitment strategies implemented, what challenges were encountered, and what solutions were devised. Recruitment strategies included partnering with community clinics, contacting eligible potential participants with trained study staff, recruiting over the summer, flyering in targeted areas of Harlem, providing a convenient study location, monetary incentives, maintaining a unified communication system (using a single phone number and simplified email address for the study), and having ongoing quality improvement processes. Challenges with our recruitment strategies included inaccurate self-reported height and weight measurements, BMI in electronic record not current, difficulty reaching potential participants (due to outdated contact information from community clinic lists), frequent no-shows and cancelations of baseline visits, and flyers were frequently torn down. Through ongoing quality improvement processes, we implemented several solutions which included the following: conducted additional community outreach (tabling at community events and clinics), partnered with community programs and institutions, expanded the eligibility criteria, utilized snowball recruitment, extended recruitment period into the fall (took advantage of back-to-school events), enhanced communication (texting more frequently, appointment reminders), and increased time spent on flyering.

**Table 3:** Intervention INC recruitment strategies and results

Strategy	Challenge(s)	Solution(s)
Obtained lists of eligible patients • Built upon existing partnerships with community clinics to obtain potential study participants • Partnered with FQHC to obtain potential study participants	• Difficulty receiving approvals to obtain lists in secure way • Difficulty creating broad enough eligibility criteria to identify patients • Difficulty with accurate databases to identify patients • Unable to obtain patient list from FQHC in timely manner due to privacy concerns	• Additional community outreach—tabled at community events, school events, and community clinics locations • Provided nutritional education • Provided food samples • Partnered with community programs and institutions—elementary/junior high schools, public parks, YMCA
Contacted eligible patients with trained study staff (including bilingual study staff) • Sent letters with study information to potential participants from patient lists (selected based on BMI measure from last medical visit) and co-signed by clinic physician • Cold called from patient lists	• Difficulty in reaching potential participants from community clinic lists due to outdated contact information • Unable to recruit participants within target time frames • Frequent no-shows and cancelations of baseline visits • Parents provided inaccurate estimates of child’s height and weight • Difficulty recruiting children in overweight/obese BMI percentile categories	• Expanded BMI eligibility criteria • Conducted snowball recruitment—sought referrals from parental participants • Extended recruitment period • Required potential participants’ verbal confirmation for 1- and 3-day confirmation • Enhanced communication, i.e., texting more frequently
• Provided a family-friendly experience • Positive interactions with study staff	• Additional children and family members attended study visits • Not enough snacks for additional people • Initially, no methods of entertaining/distracting additional kids • Additional people in the room distracted parents/kids during data collection	• Provided entertainment for other siblings, i.e., toys, drawing • Provided access to wifi (to use on personal devices) for older siblings/other adults • Ensured availability of larger meeting space or multiple spaces if multiple people came • Bought additional supplies
• Recruited in the summer when families have more time	• Slow recruitment	• Extended recruitment into the fall and took advantage of back-to-school events and fall festivals • Extended available times for data collection sections to include after-school hours and evenings
• Flyered in targeted areas of Harlem (included bilingual study staff, always in pairs)	• Community push-back • Flyers frequently torn down	• Increased time spent on community flyering/increase number of flyers posted • More strategic flyering (e.g., posted in local businesses with their approval, distributed flyers to interested local organizations such as churches, clinics)
• Convenient study location centrally located in East Harlem neighborhood and flexible study visit dates/times (included weekends and evenings)	• Unanticipated issues with allowing study participants to enter building (with security)	• Enhanced communication with building security • Requested parents to text us directly upon arrival and did not rely on security to call study staff
Incentives • Up to $70 in gift cards for the parents/guardians for completing study visit • Up to $65 in gift cards for children completing study visits • $100 gift card raffle entry for dyad participants that completed all study components • Variety of gift cards to choose from: Aldi, Wholefoods, Modell’s, Target		
Unified communication system • Used Google Voice number as single study phone and texting line • Single Hunter College email address accessible to all study staff • Used Google calendar as central scheduling platform	• Coordinated monitoring of the study line and email (especially with Spanish-speaking participants) • Coordinated availability of study staff to moderate study visit (originally the person who made contact would also be moderator, but changed to whoever was available to moderator session)	• Additional staff (especially Spanish-speaking) added to the study team • Standardized monitoring/scheduling procedure was incorporated

### Retention

#### Study implementation

Eighty-nine dyads completed all data collection activities at T1. Table 4 shows the duration of selected data collection activities. The average length of time that it took a dyad to complete an in-person session was 65 min, 73.5 min for a child to complete a phone interview, and 74 min for a parent to complete a phone interview.

**Table 4:** Duration in minutes of Intervention Inc data collection activities

		*n*	Mean (SD)	Range
T1	Overall	89	69 (14)	39–119
	Child survey	89	25 (7)	4–46
	Parent survey	89	12 (6)	4–38
T2**	Child interview	73	16 (5)	6–40
T3**	Child interview	74	16 (6)	4–37
	Parent interview	74	18 (8)	3–58
T4	Overall	72	61 (13)	39–101
	Child survey	75	19 (8)	4–40
	Parent survey	76	10 (6)	3–32
	Interview	76	19 (6)	6–33

*Independent sample *t* tests were run and there were no significant differences incompletion times between Experimental and Comparison arms

**Survey time not available because they were not completed in person

#### Participant retention

Eighty-nine (100%) dyads completed surveys at T1, 77 (86.5%) dyads at T2, 79 (88.8%) dyads at T3, and 75 (84.3%) dyads at T4. No statistically significant differences in completion among gender, race/ethnicity, group allocation, age, BMI category (child-only), household income.

#### Retention strategies and challenges

Table 5 summarizes the retention strategies used during the study, what challenges were encountered and what solutions were made. Retention strategies included assigning one study member staff to a dyad for the length of the study, incremental monetary incentives, providing a family-friendly experience (such as accommodating other family members during in-person study sessions and providing toys for younger siblings), flexible scheduling (evening and weekends), maintaining a unified communication system (using a single phone number and simplified email address for the study), and having ongoing quality improvement processes. Challenges with our retention strategies involved issues with mailing gift cards to participants, unplanned participants’ guests attending study visits, and participant issues with technology use. Through ongoing quality improvement processes, we implemented several solutions: providing availability for participants to pick up gift cards at the study site, accommodating study participants’ guests, and offering technical assistance over the phone or in-person with trained study staff.

**Table 5:** Intervention INC retention strategies and results

Strategy	Challenge(s)	Solution(s)
• Assigned trained study staff to dyads at baseline visit for the length of the study	• Study staff left while the study was still in progress	• Study staff that were leaving gave notice to PI • Reassigned dyads to available trained study staff
Incentives • Up to $70 in gift cards for the parents/guardians for completing study visit. Amount increased for each study visit • Up to $65 in gift cards for children completing study visits. Amount increased for each study visit. • $100 gift card raffle entry for dyad participants that completed all study components. • Variety of gift cards to choose from: Aldi, Wholefoods, Modell’s, Target o ○ Based on previous research	Mailing gift cards • Due to mailbox issues, some participants did not receive gift cards	• Participants that were unable to receive mail picked up gift cards at the study site
Family-friendly experience and interactions with study staff • No explicit rule around bringing additional family members to study visit	• Additional children/members attended study visits • Initially, not enough snacks for additional people • Initially, no methods of entertaining additional kids • Having additional people in the room distracted parents/kids during data collection	• Provided entertainment for other siblings, i.e., toys, drawing • Provided access to wifi (to use on personal devices) for older siblings/other adults • Ensured availability of larger meeting space or multiple spaces if multiple people came
Flexible options for completing second and third study visits • Telephone • Video calling	• Some adult participants were unable to complete questionnaires on their own because they were not comfortable using technology	• Participants were offered the option to come in person and meet with trained study staff to complete the questionnaire • Participants were offered assistance completing the questionnaire through the phone
Provided a hospitality room and were greeted by trained study staff • Toys • Food and beverages • Seating area		
Internal communication and ongoing quality improvement • Weekly meeting to discuss challenges • Trained in providing excellent customer services ○ Empathy ○ Non-judgmental ○ Non-confrontational ○ Culturally sensitive		
Convenient study location centrally located in East Harlem neighborhood and flexible study visit dates/times (included weekends and evenings)		
Unified communication system • Google Voice number as single study phone and texting line • Single Hunter College email address accessible to all study staff • Google calendar as central scheduling platform	• Coordinated monitoring of the study line and email (especially with Spanish-speaking participants)	• Additional staff (especially Spanish-speaking) were added to the study team • Standardized monitoring/scheduling procedure incorporated
• Thank you cards after third study visit and reminder for last in-person study visit • 2 recipes sent between third study and last in-person study visit		

#### Gift cards

Across all time points, most children selected the large department store (79.8%), followed by the sporting goods store (15.3%), the supermarket chain specializing in selling organic products (4.7%), and the discount supermarket chain (0.3%). Parents selected gift cards for T1, T3, and T4 and across these time points most parents selected the large department store (73.1%), followed by the supermarket chain specializing in selling organic products (13.5%), the discount supermarket chain (7.8%), and the sporting goods store (5.7%).

#### Study experience

Seventy-six adults and 75 child participants completed the online study experience survey at T4. Of the 76 adults that completed the survey, 97.4% felt that they received enough information about the study, 98.4% felt that study staff answered their questions properly, and 97.4% felt that study staff were flexible in scheduling your interview calls and visits. Of the 75 child participants that completed the survey, 68% were very satisfied/satisfied with their experience in the study. Additionally, 81.4% were extremely satisfied/satisfied with how study staff communicated and interacted with them. There are no statistically significant differences in the study experience responses between the experimental arm and the comparison arm for both adult and child participants.

## Discussion

Recruitment and retention of study participants, especially from low-income, minority populations, has historically been a significant challenge [15, 16]. Few studies, furthermore, provide reliable data on recruitment and retention of low-income, minority children [6]. Intervention INC successfully recruited 89 parent-child dyads over the course of 4 months and retained 84.3% of dyads during its 4.5-month study using a variety of strategies.

### Recruitment

In this study, the use of multiple recruitment strategies and continuous assessment of and adjustment to recruitment needs increased the rate of enrollment over time. In the early phase of recruitment (August 2017), recruitment strategies were largely limited to mailing letters and calling individuals identified by the CBO health clinic, and random flyering in the East Harlem community. Dyad enrollment was relatively low during this month and thus required a re-assessment and adjustment of approaches used. Coinciding with back-to-school and Fall community and school events, additional recruitment activities were implemented, such as attending and tabling at large-attendee school/community events. Additionally, targeted flyering was done near local businesses and through partnerships with community organizations, and event tabling was utilized. Consequently, the rate of dyad enrollment was faster from September to October 2017.

Interestingly, despite having access to potential participant list of children that had used the services of a CBO health clinic within the last 2 years and sending recruitment letters signed by the CBO Medical Director, a CBO Pediatrician, and the study Principal Investigator, few participants were enrolled using this approach. Only 12.4% of enrolled participants learned about the study through community clinic partnerships, which included the CBO potential participant lists. By comparison, the most successful recruitment strategies included community flyering and attending community events as 33.7% and 39.3%, respectively, of our study participants were enrolled by these strategies.

Aligned with the recruitment experiences in this study, Hartlieb et al. found in their study of recruiting minority adolescents with obesity that the use of multiple recruitment strategies—including identifying eligible participants through clinical partnerships, flyering within the community, and participating in health fairs—is beneficial and can yield high retention [11]. The decision to enhance community-based recruitment may have accelerated enrollment since individual choices are often dependent upon strong community ties [6]. Community-based recruitment of minority participants also likely creates trust and more positive relationships with study staff [17]. Indeed in other literature, recruitment of low-income, minority populations have most often included methods such as advertisements, targeted mailing and calls using organization lists, neighborhood canvassing, and community and health fair presentations [18].

It should be noted that our ability to continuously assess and modify strategies may have been, in large part, key to our recruitment success. At weekly team meetings, we were able to evaluate the data and therefore quickly implement new strategies as needed. For example, upon realizing that the BMI percentile criteria was too limited, we subsequently modified it to be more inclusive. Additionally, our staff members were flexible and allowed participants to schedule baseline appointments on weekends and during late hours. Staff members were also able to accommodate a longer recruitment period that had been extended for an additional 2 months.

Similar to our experiences with accommodation and adaptability in recruitment, Warner et al. found in their study that consistent evaluation of recruitment data to create new strategies followed by quick implementation was largely attributed to their success in participant enrollment [19]. Furthermore, their study staff’s willingness to meet participant needs and general flexibility were factors also associated with successful recruitment [19]. Offering more flexible hours helps to meet the needs of individual participants; this is especially important for low-income, minority parents who are not able to compromise their work schedule in order to be part of the intervention [20].

### Retention

Retention remained high throughout the study with completion of T4 at 84.3%. No statistically significant difference was found with retention among the different demographic groupings (race/ethnicity, income, BMI category, gender, and age). These findings are in line with a similar 9-month study among racial/ethnic minority family dyads that reported 88% retention [17]. Parent and child participants reported high satisfaction rates at the end of our study. This may indicate that our retention strategies were successful in retaining Black and Latino families in East Harlem regardless of subgroups. It took participants an average of 69 min/time point to complete data collection study activities over a period of 4.5 months. The amount of time that participants commit to the research study has been one of the components used by other studies to measure participant burden [21, 22], We did not measure burden in this study, thus cannot discern if the amount of time that participants spent on completing study activities impacted retention. However, perceived participant burden is an important aspect of recruitment and retention but there have been few studies that have tried to understand participant burden or benefit. Ulrich et al. found that the higher the perceived burden of the study, the more likely participants are to think about dropping out [23]. Lingler et al. found that participation burden was inversely associated with enrolling in the study and that research burden increased as the study risk categorization increased [21]. Bodart et al. found that the length of time and time of day were important factors in perceived burden to complete study questionnaires [22]. Participants in our study reported high satisfaction rates which may indicate that they perceived the study to be low burden. However, participation burden is complex and can be associated with other factors including sociodemographic, level of trust in researchers, and perceived benefit to self or to society.

The selection of gift cards provided to families may have also led to higher retention rates. Formative research was conducted to identify appropriate stores. The large department store retailer was the most popular gift card choice among both parents and children as it had a vast array of items available. It is important to consider the versatility of the incentives provided to participants especially in low resourced areas. Additionally, successful retention strategies included flexible hours, bilingual study staff, incremental monetary incentives, metrocards, and ongoing communication with study participants. Other retention and recruitment research of racial and ethnic minority populations has indicated that these are crucial elements to a successful retention strategy [17, 24–26]. Parents indicated that they felt well informed about the study and felt that study staff answered their questions. We experienced some challenges throughout the study with retention including no-shows and technological challenges experienced by participants such as forgetting passwords/usernames and navigating the intervention tool. Our weekly staff meetings allowed us to identify these challenges early on and implement solutions to maintain high retention rates.

### Strengths/limitations of the study

This study has several limitations that should be taken into consideration. First, our findings are specific to Latino and Black families in East Harlem, NY and may not be generalizable to other ethnic/racial minorities or other geographic locations. Second, families interested in nutrition may more actively seek enrollment into a nutrition study and may be more motivated to complete the study than families that are not interested in nutrition. Third, due to the design of the study, we are unable to determine which retention strategies were most successful. Strengths of this study included detailed documentation of all study protocols, a centralized electronic communication system, and weekly meetings. These factors allowed us to have a clear understanding of protocols that were in place and allowed us to implement solutions based on participant and study staff feedback we reviewed during weekly meetings. The study was also structured to be flexible to make changes to the study protocols in response to challenges encountered throughout the study. Study staff was trained to expect changes and to bring up concerns to team meetings. Study staff also had community outreach and nutrition experience which allowed us to utilize community engagement strategies. In addition, though our findings are limited to only Latino and Black families in East Harlem, we have identified successful strategies for this specific high-risk population and potentially similar others.

## Conclusion

Intervention INC successfully recruited and retained low-income, minority children and their parents in East Harlem. Within our study, the most successful recruitment strategies included community flyering and attending community events. Our high retention rate shows that participation of minority families is possible in longitudinal studies despite a plethora of barriers to low-income, minority individuals, and potential participant burden. To effectively recruit and retain Latino and Black families into health promotion studies, our results suggest that a *variety* of strategies—particularly those that build trust and relationships—is necessary. Furthermore, implementing these various types of strategies requires careful planning, detailed tracking, frequent check-ins, and adaptability to modify as needed. More research is needed to determine specifically which recruitment and retention strategies are the most successful among Latino and Black families. This information could create more targeted strategy implementation and subsequent high levels of retention of minority populations in future interventions.

## Data Availability

The datasets used and/or analyzed during the current study are available from the corresponding author on reasonable request.

## Abbreviations

AA: African American
BMI: Body mass index (height (m)/weight (kg)^2^)
C: Control
CBO: Community-based organization
CONSORT: Consolidated Standards for Reporting Trials
E: Experimental
GED: General education development
Intervention INC: Interactive Nutrition Comics for Urban Minority Youth
mHealth: Mobile Health
*N*: Number
NY: New York
NYC: New York City
SNAP: Supplemental Nutrition Assistance Program
SPSS: Statistical Package for the Social Sciences
T1: Baseline
T2: Intervention midpoint or 3 weeks post-baseline
T3: Intervention end or 6 weeks post-baseline
T4: 3 month follow-up post-intervention
US: Unites States
